# Mammalian and bacterial adaptors function as co-disinhibitory pairs to activate the E3 ubiquitin ligase WWP2

**DOI:** 10.1016/j.jbc.2025.110847

**Published:** 2025-10-22

**Authors:** Samkeliso V. Blundell, Mei Liu, Romina Tocci, Andrea Majstorovic, Solène Tsang, David W. Holden

**Affiliations:** Centre for Bacterial Resistance Biology, Imperial College London, London, United Kingdom

**Keywords:** *Salmonella enterica*, virulence factor, E3 ubiquitin ligase, adaptor protein, ubiquitin

## Abstract

The NEDD4-like E3 ubiquitin ligase, WWP2, is involved in a range of host processes from cell differentiation to T cell immunity. Ligase activity is tightly regulated, with WWP2 being held in an autoinhibited state. The binding of a PY motif-containing adaptor, an Ndfip, *via* the WW domains of NEDD4-like E3 ubiquitin ligases leads to their disinhibition. Here, we show that the canonical Ndfip, NDFIP2, requires multiple PY motifs for interaction with and activation of WWP2. In contrast, the single PY-motif containing Ndfips TMEM127 and SUSD6 functions as a co-disinhibitory pair. TMEM127 and the *Salmonella* protein SteD also function as a co-disinhibitory pair. However, SteD requires a different region of WWP2, the C2 domain, for interaction with WWP2, and this interaction results in disinhibition of WWP2. These findings demonstrate a range of ways that Ndfips can disinhibit WWP2. To our knowledge, these are the first examples of two Ndfips functioning as co-disinhibitory pairs, and of a bacterial effector that disinhibits an E3 ubiquitin ligase.

The NEDD4-like E3 ubiquitin ligases are a family of C-terminal catalytic homologous to E6 COOH (HECT) proteins that catalyze the ubiquitination of themselves and their substrates. They are involved in a wide range of cellular processes, such as the cell cycle, cell differentiation, T cell immunity and viral egress (1, 2, 3, 4, 5). Their dysregulation can lead to oncogenesis and autoimmunity, highlighting the importance of precise regulation (6, 7). They share a common domain architecture comprising an N-terminal C2 domain, two to four tryptophan-tryptophan (WW) domains and a HECT domain (8, 9). Ligase activity is tightly regulated through auto-inhibition, with disinhibition occurring following binding of an adaptor, binding of calcium or phosphorylation of the E3 ligase (10, 11, 12). Other factors also contribute to adaptor-mediated NEDD4-like E3 ubiquitin ligase disinhibition, including membrane tethering, clustering and membrane curvature (13, 14). In adaptor-mediated disinhibition, PY motifs (proline-tyrosine motif - a term used here to denote either PPxY, or with one or two prolines in any of the −1, −2 or −3 positions with respect to the tyrosine) within NEDD4 family interacting proteins (Ndfips) interact with ligase WW domains and recruit the ligase to the target protein. Multiple PY-WW domain interactions between either of the canonical adaptors(NDFIP1 or NDFIP2) and the NEDD4-like E3 ubiquitin ligase Itch lead to the release of auto-inhibitory interactions and subsequent disinhibition of Itch (11).

The NEDD4-like E3 ubiquitin ligase, WWP2, is co-opted by *Salmonella* effector protein SteD, which is translocated into mammalian cells by the *Salmonella* pathogenicity island-2 (SPI-2) type III injectisome. In antigen-presenting cells, SteD functions *via* an integral membrane adaptor protein called TMEM127 and WWP2 to deplete mature peptide-loaded Major Histocompatibility Complex class II (mMHCII) molecules, CD97 and B7.2/CD86 from the plasma membrane, thereby inhibiting T cell activation and proliferation (15, 16, 17). SteD interacts with TMEM127 *via* amino acids within the SteD transmembrane regions, and TMEM127 interacts with WWP2 *via* the C-terminal PPxY motif of TMEM127: this showed that TMEM127 is an Ndfip (16). By interacting with both TMEM127 and mMHCII or CD97, SteD induces WWP2-dependent ubiquitination of amino acids in the cytoplasmic regions of mMHCII and CD97, leading to their lysosomal degradation (16, 17).

Although the interactions between TMEM127, WWP2 and SteD (15, 16) are sufficient to explain how WWP2 comes into close proximity with MHCII, through recruitment by TMEM127 (16), it is not clear how WWP2 is disinhibited. Interestingly, both TMEM127 and WWP2 were recently shown to deplete surface MHCI in acute myeloid leukemia (AML) cells. In this case, another mammalian protein, SUSD6, which interacts with MHCI and TMEM127, was found to be a component of the TMEM127/WWP2 complex (18). This raised the possibility that SUSD6 and SteD function with TMEM127 in an analogous fashion. In this study, we used mutagenesis, together with cellular binding and disinhibition assays, to shed more light on the mechanism by which WWP2 is disinhibited by a canonical Ndfip (NDFIP2), SUSD6 and SteD.

## Results

### WW2 is required for the WWP2-NDFIP2 interaction

**Figure 1:**
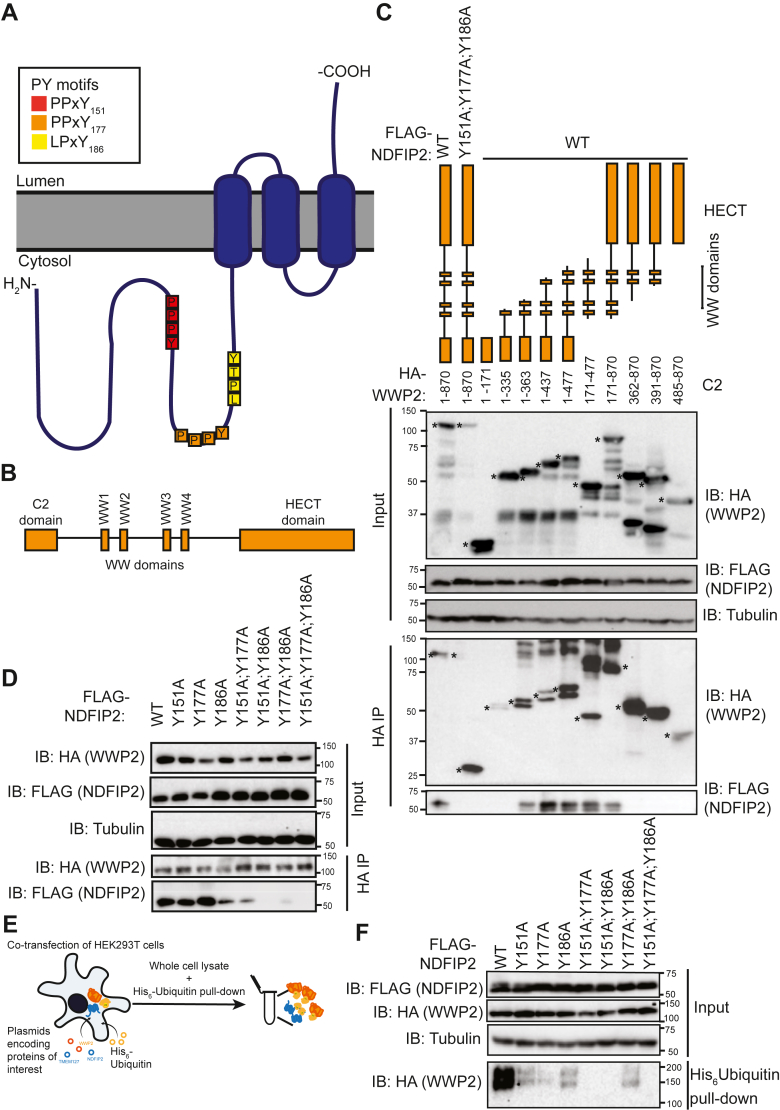
**NDFIP2 interacts with the WW2 domain of WWP2 and requires its three PY motifs to interact with and disinhibit WWP2.***A*, Cartoon of NDFIP2 topology and PY motifs. *B*, Schematic of WWP2 domains. *C and D*, HEK293T cell transfection with FLAG-NDFIP2 (WT or mutant) and HA-WWP2 (full length or truncation as indicated), followed by HA-immunoprecipitation and immunoblot of immunoprecipitate (IP). ∗ Indicates relevant bands. *E*, Schematic of the His_6_-ubiquitin pull-down assay used in *F*. *F*, HEK293T cell transfection with FLAG-NDFIP2 (WT or PY mutant), HA-WWP2 and His_6_-ubiquitin, followed by His pulldown and immunoblot of pulldown. All blots are representative of three independent experiments. PY, proline-tyrosine motif.

Although WW domains have the greatest affinity for PY motifs in which a tyrosine is preceded by two prolines in the −2 and −3 positions, they can interact with PY motifs that have one or two prolines in any of the −1, −2 or −3 positions (19). NDFIP2 is a canonical Ndfip that disinhibits WWP2 (11)Mund and Pelham, 2009), but the interaction between its three PY motifs and WWP2 has not been reported previously. The first and second PY motifs are of the form PPxY, and the third corresponds to LPxY (Fig. 1*A*). First, to determine which WWP2 domains (Fig. 1*B*) are required for the NDFIP2-WWP2 interaction, we constructed 10 truncations of WWP2 extending from either N- or C-termini containing or lacking the C2, WW1, WW2, WW3, WW4, and HECT domains (Fig. 1*C*). These were based on previously published truncations that assessed WWP2 disinhibition (10, 20). NDFIP2 only interacted with WWP2 truncations that contained the WW2 domain (Fig. 1*C*).

### NDFIP2 requires all three PY motifs for interaction with and disinhibition of WWP2

Single, double and triple tyrosine-for-alanine substitution mutants affecting the three NDFIP2 PY motifs were constructed (Fig. S1) and assessed for their ability to interact with or disinhibit WWP2. The WWP2-NDFIP2^Y151A^ (PY1-mutated) and WWP2-NFDIP2^Y177A^ (PY2-mutated) interactions were similar to the WWP2-^NDFIPWT^ interaction, whereas the WWP2-NDFIP2^Y186A^ (PY3-mutated) interaction was weaker and similar to that of the WWP2-NDFIP2^Y151A;Y177A^ interaction (Fig. 1*D*). NDFIP2^Y15A;Y186A^ and NDFIP2^Y151A;Y186A^ had a greatly reduced interaction with WWP2 compared to NDFIP2^WT^ (Fig. 1*D*). Therefore, Y186 of NDFIP2 has the greatest contribution of the three PY motif tyrosines to the interaction with WWP2.

We then assessed the contribution of the PY motifs to disinhibition of WWP2 using a well-established *in cellulo* ubiquitination assay in HEK293T cells (11, 13, 21) (Fig. 1*E*). When WWP2 is disinhibited, it auto-ubiquitinates its HECT domain. Therefore, disinhibition can be assayed by monitoring the ubiquitination of WWP2 (22). Here, WWP2 and His_6_-tagged ubiquitin were co-expressed with either NDFIP2 (wild-type or mutant as indicated). His pull-down was then used to assess ubiquitinated proteins. In agreement with previous findings (11), NDFIP2^WT^ induced ubiquitination of WWP2 and NDFIP2^Y151A;Y177A;Y186A^ did not (Fig. 1*F*). All single PY motif mutants showed markedly reduced disinhibition of WWP2 compared to wild type. NDFIP2^Y151A;Y177A^ and NDFIP2^Y151;Y186A^ were unable to disinhibit WWP2 (Fig. 1*F*). This suggests that at least Y151, in addition to either Y177 or Y186 are required for disinhibition. Evidently, although Y151 and Y177 are not required for binding of NDFIP2 to WWP2, they contribute to the NDFIP2-WWP2 interaction and are important for subsequent WWP2 disinhibition. While all three tyrosines appear to be important, Y186 and Y151 contribute most to binding and disinhibition, respectively.

### A single PY motif within TMEM127 is required for interaction with WWP2

**Figure 2:**
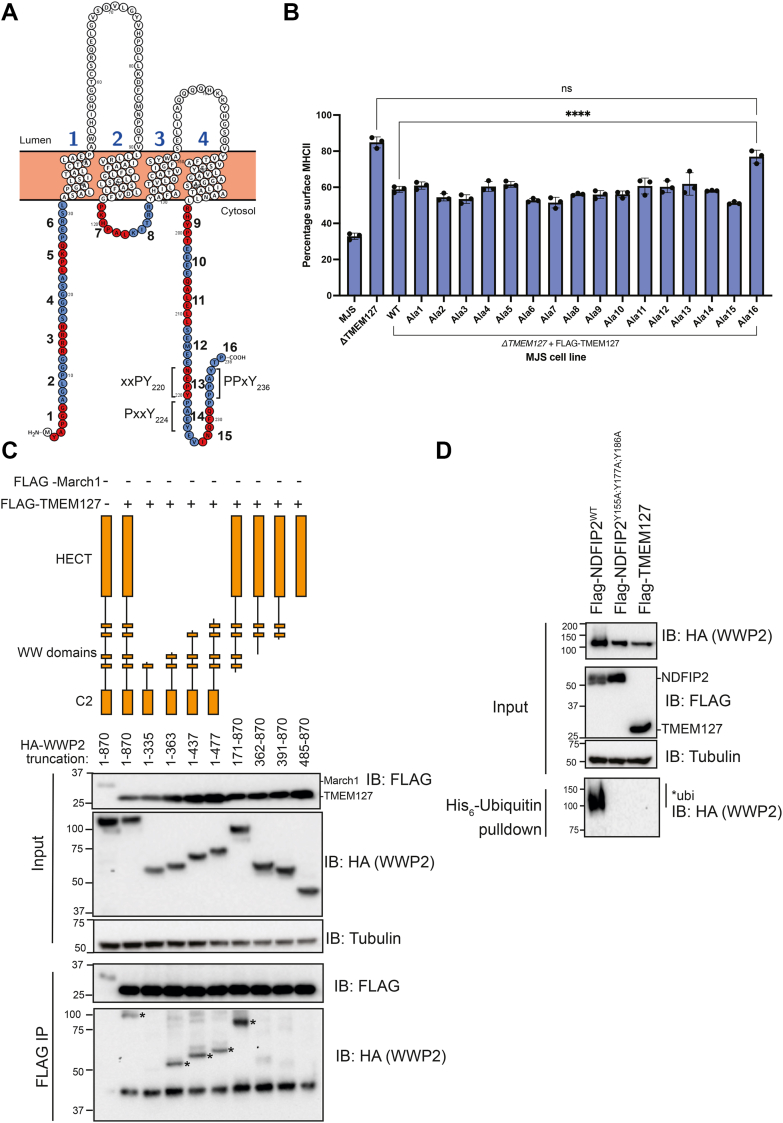
**TMEM127 is an atypical non-activating Ndfip of WWP2 and a single PY motif is required for interaction with WWP2.***A*, Protter image of TMEM127 Alanine mutants highlighted in alternating *red* and *blue* (numbered). *B*, mMHCII surface levels of Mel JuSo cells (wild-type or mutant as indicated) infected with *Salmonella*. Cells were analysed by flow cytometry and the data represent geometric mean fluorescence intensity of surface level mMHCII signal of infected cells as a percentage of non-infected cells. Means ± SD from three independent experiments with each data point shown being the value obtained from each independent experiment. Data were analysed using a one-way ANOVA with a Tukey’s correction for multiple comparisons. N = 3. ∗∗∗∗*p* < 0.0001 *C*. HEK293T cell transfection with FLAG-March1 or FLAG-TMEM127 and HA-WWP2 (full length or truncation), followed by HA-immunoprecipitation and immunoblot of immunoprecipitate (IP). ∗ Indicates relevant bands. *D*, HEK293T cell transfection with FLAG-NDFIP2^WT^, FLAG-NDFIP2^Y151A;Y177A;Y186A^ or FLAG-TMEM127, HA-WWP2 and His_6_-ubiquitin, followed His-pull down, and analysis of pulldown by immunoblotting. All blots are representative of three independent repeats.

TMEM127 has three putative PY motifs (Fig. 2*A*): xxPY_220_ of unknown function, PxxY_224_, which together with PPxY_236_ mediates degradation of the tyrosine kinase receptor RET by NEDD4 (23). PPxY_236_ is also required for TMEM127 for the interaction with either WWP2 or NEDD4 (16, 23). We first analysed the contribution of each PY motif and the cytoplasmic regions of TMEM127 to SteD-mediated reduction of surface levels of mMHCII during *Salmonella* infection by scanning mutagenesis. Testing of 16 different alanine substitution mutants (Ala1-16) (Fig. 2*A*), each expressed in TMEM127 knockout Mel Juso cells (Fig. S2*A*), revealed only one cytoplasmic region of TMEM127 required for SteD-mediated reduction of surface levels of mMHCII – the Ala16 region (Fig. 2*B*). This region contains the PPxY_236_ motif (16). Even though the putative PY motifs mutated in Ala13 and Ala14 did not contribute to SteD-mediated reduction of surface levels of mMHCII (Fig. 2*B*), they might participate in ligase interaction. To test this, single, double and triple point mutations of the tyrosine residue within each putative PY motif and the known PPxY motif were generated (Fig. S2*B*) and assessed for their interaction with WWP2. Mutation of Y236 to alanine diminished interaction between TMEM127 and WWP2 with no additive effect upon additional mutation of Y220 and/or Y224 (Fig. S2*C*). Therefore, TMEM127 has a single PY motif (PPxY_236_), required for interaction with WWP2.

### TMEM127 is an atypical non-activating Ndfip of WWP2

Next, we determined the WWP2 domains required for the TMEM127-WWP2 interaction. FLAG-immunoprecipitation of FLAG-TMEM127 pulled down WWP2 truncations containing the WW2 domain (Fig. 2*C*). Therefore, a strong interaction between WWP2 and TMEM127 requires the presence of the WW2 domain of WWP2 and the PPxY_236_ motif of TMEM127 (Fig. 2*C*), suggesting that these might be principal interacting regions. We then assessed whether TMEM127 could disinhibit WWP2 in the *in cellulo* disinhibition assay. Unlike NDFIP2, TMEM127 failed to induce ubiquitination of WWP2 (Fig. 2*D*), suggesting that it is an atypical Ndfip.

### SUSD6 requires its LPxY motif for interaction with WWP2, and together SUSD6 and TMEM127 co-disinhibit WWP2

**Figure 3:**
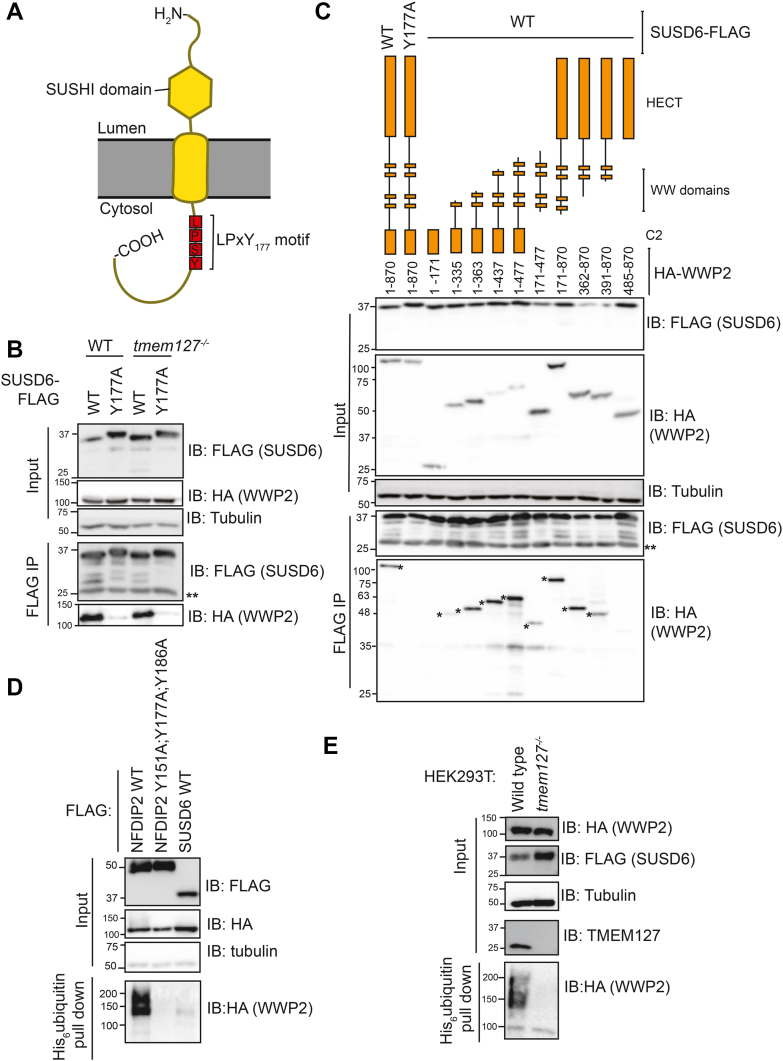
**SUSD6 interaction with and disinhibition of WWP2.***A*, cartoon of SUSD6 topology and LPxY motif. *B* and *C*, HEK293T (WT or TMEM127^−/−^) cell transfection with SUSD6-FLAG (WT or Y177A) and HA-WWP2 (full length or truncation) followed by FLAG-immunoprecipitation and immunoblot of immunoprecipitate (IP). ∗Indicates relevant bands. *D* and *E*, HEK293T (WT or TMEM127^−/−^) cell transfection with FLAG-NDFIP2 (WT or PY mutant) or SUS6-FLAG, HA-WWP2 and His_6_-ubiquitin followed by His pull down and analysis of pulldown by immunoblotting. All blots are representative of three independent experiments.∗∗indicates light chain.

TMEM127 interacts with an endogenous transmembrane protein, SUSD6, and WWP2 to mediate the reduction of MHCI in tumour cells (18). SUSD6 has one transmembrane domain and within the cytoplasmic region, there is a possible LPxY motif (L_174_P_175_S_176_Y_177_) (Fig. 3*A*). Therefore, we reasoned that SUSD6 might function as an Ndfip of WWP2 and regulate its activity. The WWP2-SUSD6 interaction was dependent on the presence of Y177 within the LPxY motif and did not require TMEM127 (Figs 3*B* and S3, *A*–*D*). Therefore, like TMEM127, SUSD6 is an Ndfip. Although two Ndfips have not previously been shown to function simultaneously on the same NEDD4-like E3 ubiquitin ligase, both SUSD6 and TMEM127 are required for SUSD6-mediated reduction of surface MHCI (18). Therefore, we hypothesized that SUSD6 and TMEM127 might cooperate by interacting with different WW domains of WWP2. To investigate this, we analyzed the domains of WWP2 that are required for the WWP2-SUSD6 interaction. In addition to an interaction between SUSD6 and WWP2 truncations containing the WW2 domain (as for TMEM127 and NDFIP2), there was also an interaction between SUSD6 and WWP2 truncations containing the WW3/WW4 domains (Fig. 3*C*).

We then determined if SUSD6 could disinhibit WWP2. In the presence of TMEM127, SUSD6 induced weak ubiquitination of WWP2 (Fig. 3*D*). This was less than the amount of ubiquitination induced by NDFIP2^WT^; NDFIP2^Y151A;Y177A;Y186A^ did not induce ubiquitination of WWP2 (Fig. 3*D*). In the absence of TMEM127, SUSD6 failed to induce ubiquitination of WWP2 (Fig. 3*E*). Together, these results show that SUSD6 and TMEM127 co-disinhibit WWP2.

### SteD and TMEM127 co-disinhibit WWP2

**Figure 4:**
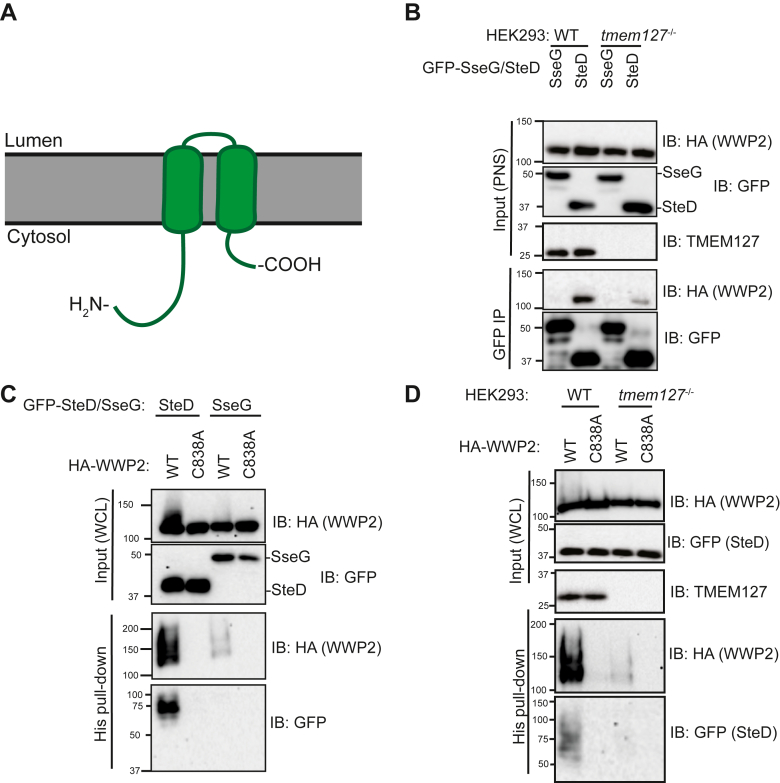
**SteD is an NDFIP and TMEM127 and SteD are co-disinhibitors of WWP2.***A*, cartoon of SteD topology. *B*, HEK239T cell (WT or *tmem127*^−/−^) transfection with GFP-SseG or GFP-SteD and HA-WWP2, followed by GFP-immunoprecipitation and analysis of immunoprecipitate (IP) by immunoblotting. *C* and *D*, HEK293T cells (WT or *tmem127*^−/−^) transfection with GFP-SseG or GFP-SteD, HA-WWP2 (WT or C383A), followed by His pulldown and analysis of pulldown by immunoblotting. Post-nuclear supernatant (PNS); whole cell lysate (WCL). All blots are representative of three independent experiments.

Having established the regions required by three endogenous transmembrane Ndfips (NDFIP2, TMEM127 and SUSD6) for interaction with and disinhibition of WWP2, we investigated how the bacterial effector and transmembrane protein SteD (Fig. 4*A*) contributes to WWP2 function. First, we determined if there is a SteD-WWP2 interaction and, if so, whether this interaction requires TMEM127. Immunoprecipitation of GFP-SteD from transfected HEK293 cells showed that WWP2 co-immunoprecipitated with GFP-SteD. In the absence of TMEM127, the amount of WWP2 co-immunoprecipitated with SteD was reduced but not eliminated (Fig. 4*B*).

Given that SteD functions with TMEM127 to reduce surface levels of MHCII in a way that is analogous to SUSD6/TMEM127/WWP2-mediated reduction of surface levels of MHCI (16, 18), we hypothesized that SteD might disinhibit WWP2. Using the His_6_-ubiquitin pull-down assay, we compared WWP2 ubiquitination by SteD with that of another integral membrane effector of unrelated function, SseG. Like SteD, SseG is an SPI-2 injectisome effector that is translocated into host cell membranes; however, SseG is required for the localisation of *Salmonella*-containing vacuoles to the Golgi network (24) through interaction with the Golgi-network-associated protein ACBD3 (25). We found that SteD induced much stronger ubiquitination of WWP2 than SseG (Fig. 4*C*). The very weak disinhibition of WWP2 in cells expressing SseG (Fig. 4*C*) is presumably caused by other endogenous Ndfips in HEK293T cells. SteD also undergoes ubiquitination, and this is important for its function in reducing mMHCII surface levels (16). Therefore, we used a catalytic point mutant of WWP2 (WWP2^C838A^) to determine whether SteD-induced ubiquitination of WWP2 was auto-ubiquitination, and if ubiquitination of SteD was caused by catalytic activity of WWP2 (Fig. 4*C*). SteD-induced WWP2 ubiquitination required the catalytic activity of WWP2, and the ubiquitination of SteD itself also required WWP2 catalytic activity (Fig. 4*C*). These results reveal that SteD disinhibits WWP2 and that SteD is ubiquitinated by WWP2.

SteD requires TMEM127 for SteD-mediated ubiquitination of mMHCII (16). Therefore, we investigated the level of auto-ubiquitination of WWP2 induced by SteD in HEK293T cells lacking TMEM127. SteD-induced WWP2 ubiquitination was greatly diminished in TMEM127 knockouts when compared to WT cells (Fig. 4*D*). Together, these results show that, as for SUSD6 and TMEM127, SteD and TMEM127 work together as co-disinhibitors of WWP2.

### SteD requires its N and C-terminal cytosolic domains to disinhibit WWP2

**Figure 5:**
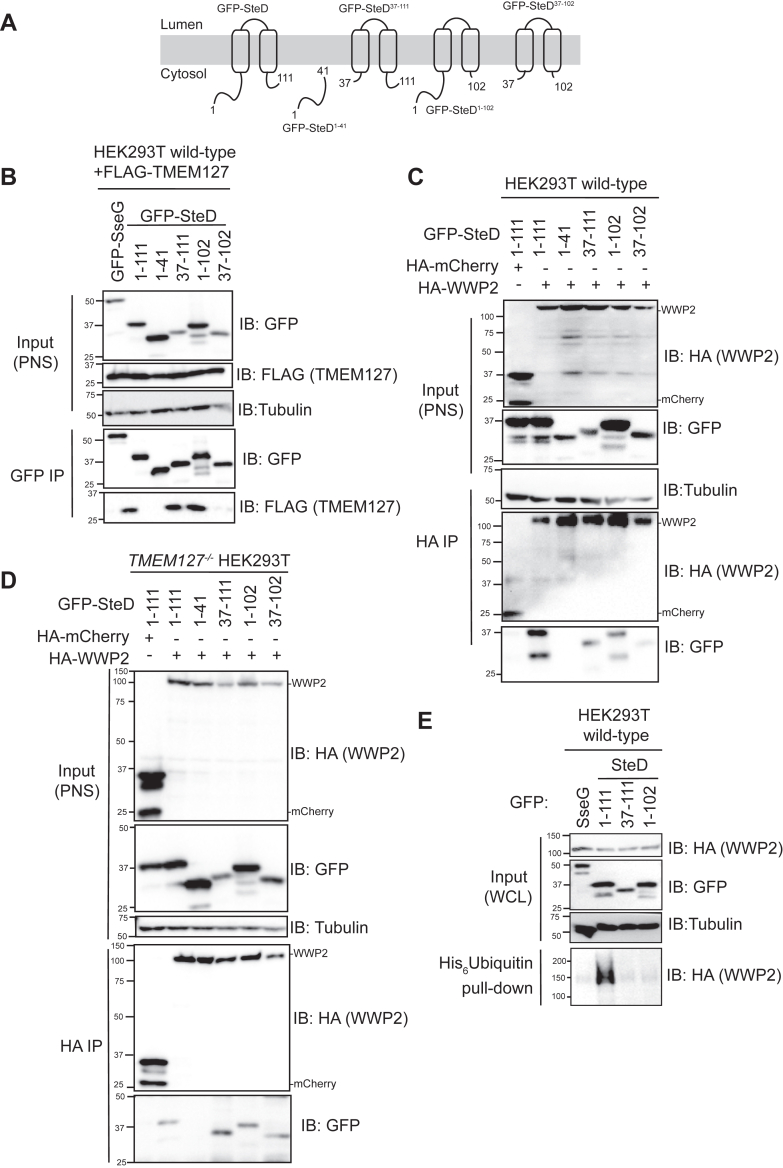
**Truncations of SteD that interact with TMEM127 and WWP2 and disinhibit.***A*, a schematic of the GFP-SteD truncations used in these experiments. *B*, HEK239T cell transfection with GFP-SteD (WT or truncation) and FLAG-TMEM127, followed by GFP-immunoprecipitation (IP) and analysis of immunoprecipitate by immunoblotting. *C & D*, HEK239T cells (*C*. wild-type, *D*. *tmem127*^−/−^) transfection with GFP-SteD (WT or truncation) and HA-WWP2, followed by HA-immunoprecipitation and analysis by immunoblotting. *E*, HEK293T cells transfection followed by His pulldown and analysis by immunoblotting. All blots are representative of at least three independent experiments. WCL, whole cell lysate. PNS, post-nuclear supernatant.

In addition to lacking a PPxY motif, SteD lacks proline-rich regions and any putative PY motifs that could interact with WW domains and contribute to its ability to disinhibit WWP2. Therefore, to determine which region/s of SteD contribute/s to the interaction with and disinhibition of WWP2, truncations of SteD were used (Figs. 5*A* and S3, *E*–*F*) (26). The interaction between these truncations and TMEM127 was assessed first. It was previously shown that mutations in the transmembrane regions of SteD can prevent the interaction with TMEM127 (16). TMEM127 co-immunoprecipitated with truncations of SteD comprising both transmembrane domains, together with either the N-terminal or C-terminal cytoplasmic domains. It did not co-immunoprecipitate with cytosolic SteD^1-41^ (the N-terminal 41 amino acid peptide), and there was little co-immunoprecipitation with SteD^37-102^ (which lacks both the 36 cytoplasmic N-terminal amino acids and nine cytoplasmic C-terminal amino acids) (Fig. 5*B*). Therefore, in addition to the transmembrane regions of SteD, the N- or C-terminal of SteD contributes to the SteD-TMEM127 interaction.

In the presence of TMEM127, SteD^37-111^, SteD^1-102,^ and SteD^37-102^ interacted with WWP2 (Fig. 5*C*). Since SteD^37-111^ and SteD^1-102^ interactions could be indirect (*via* TMEM127), we assessed SteD-WWP2 interactions in the absence of TMEM127. In the absence of TMEM127, SteD^1-111^ (full length), SteD^37-111^, SteD^1-102^ and SteD^37-102^ all interacted with WWP2 (Fig. 5*D*), which provides strong evidence for an interaction between SteD and WWP2 that does not involve TMEM127. Despite being able to bind both TMEM127 and WWP2, SteD^37-111^ and SteD^1-102^ truncations were poor disinhibitors of WWP2 (Fig. 5*E*). This suggests that the N- and C-terminal cytosolic regions of SteD are important for WWP2 disinhibition.

### The C2 domain of WWP2 is required for the WWP2-SteD interaction and contributes to disinhibition of WWP2

**Figure 6:**
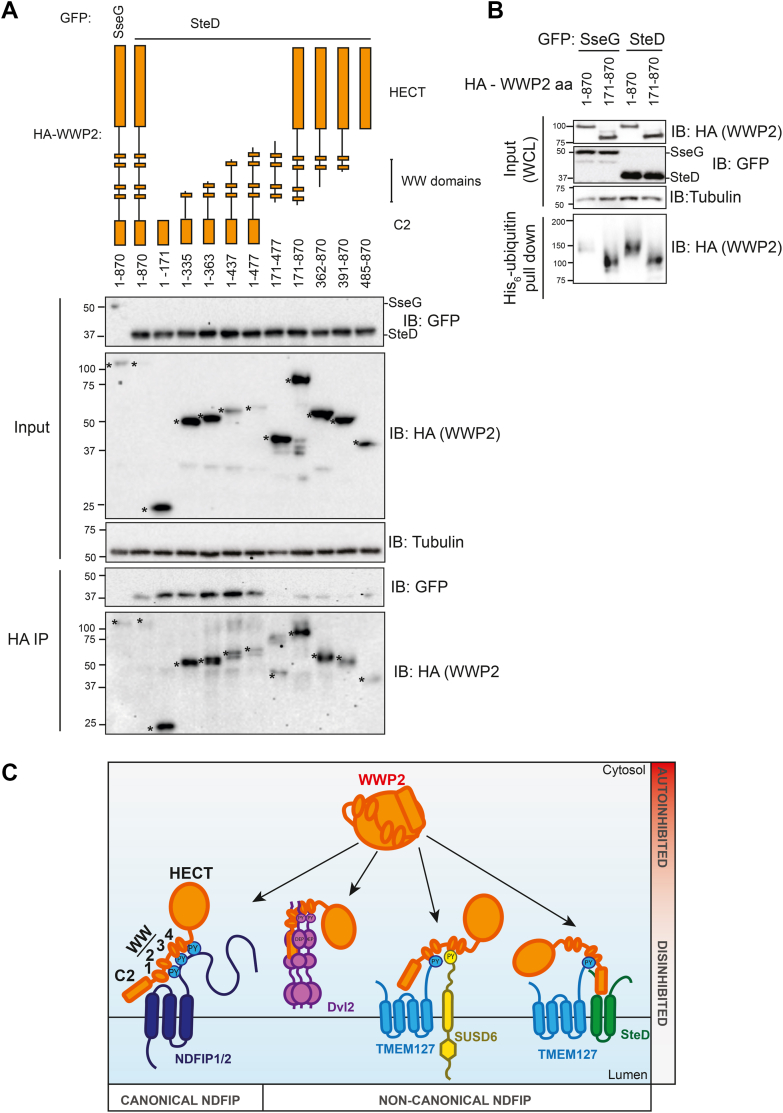
**SteD interacts with the C2 domain of WWP2, and the C2 domain contributes to disinhibition of WWP2.***A*, HEK293T cell transfection with GFP-SseG or GFP-SteD and HA-WWP2 (full length or truncation), followed by HA-immunoprecipitation (IP) and analysis by immunoblotting. ∗ Indicates relevant bands. *B*, HEK293T cell transfection followed by His pulldown and analysis by immunoblotting. *C*, model of disinhibition of WWP2 by canonical and non-canonical Ndfips. All blots are representative of three independent experiments. PY, proline-tyrosine motif.

Since SteD does not have any PY motifs, we hypothesized that a region of WWP2 other than the WW domains might be required for the WWP2-SteD interaction and that these interactions might account for how SteD disinhibits WWP2. Immunoprecipitation of HA-WWP2 truncations showed that SteD interacted with all truncations that contain the C2 domain of WWP2, and the C2 domain (amino acids 1–171) was sufficient for this interaction (Fig. 6*A*). There was also a very weak interaction between SteD and WWP2 truncations containing the HECT domain (Fig. 6*A*). Given that the WWP2-SteD interaction required domains not required for the WWP2-TMEM127 interaction (Fig. 2*C*), these C2 domain-SteD interactions might be direct.

The contribution of the C2 domain of WWP2 to its auto-inhibition is not clear. Wiesner *et al.* showed that *in vitro*, a WWP2 mutant lacking the C2 domain had a greater level of auto-ubiquitination compared to the wild-type protein (27). Conversely, Chen *et al.* showed that truncation of the C2 domain did not lead to increased auto-ubiquitination of WWP2 *in vitro* (10). We assessed the importance of the C2 domain to auto-inhibition of WWP2 with the His_6_-ubiquitin pull-down assay in HEK293T cells by comparing full-length WWP2 with WWP2 lacking the C2 domain (WWP2^171-870^). In contrast to full-length WWP2, this truncation underwent disinhibition in the presence of the control *Salmonella* effector SseG, to a level similar to that detected when SteD was expressed together with either full-length or C2-truncated WWP2 (Fig. 6*B*). Our results are therefore in agreement with Wiesner *et al.* (2017) and strongly suggest that binding of SteD to the C2 domain of WWP2 relieves its autoinhibition (Fig. 6*C*).

## Discussion

Previous studies have shown that the NEDD4-like E3 ubiquitin ligases bind to PY motifs of Ndfips, and this can lead to disinhibition of ligase activity. In this work, we expand our understanding of requirements for adaptor binding and disinhibition of WWP2 by three mammalian proteins (TMEM127, NDFIP2 and SUSD6) and one from the pathogenic bacterium *Salmonella enterica* (SteD). Our experiments suggest that TMEM127, NDFIP2 and SUSD6 all interact with the WW2 domain of WWP2 and that SUSD6 also might interact with the WW3 and WW4 domains. In contrast, our work suggests that SteD interacts with the C2 domain of WWP2. We cannot rule out the possibility that other proteins might be involved in these multi-protein complexes and that some of the interactions we detect might be indirect.

The WWP2-SUSD6 interaction does not require TMEM127, but both SteD and SUSD6 require TMEM127 to disinhibit WWP2. Therefore, SUSD6/TMEM127 and SteD/TMEM127 function as co-disinhibitor pairs, which makes them a novel class of Ndfips. Intramembrane interactions between TMEM127 and SteD (16) would enable the formation of a three-protein complex and cooperative binding to both WW2 and C2 domains, leading to relief of autoinhibition and WWP2 disinhibition. It is not known if or how SUSD6 interacts with TMEM127, but together, their combined ability to bind multiple WW domains could enable WWP2 disinhibition. As TMEM127 is a flexible Ndfip able to function with both SUSD6 or SteD, there might be other endogenous TMEM127-partnering co-disinhibitors.

One PY motif of TMEM127 is required for interaction with WWP2 (16), but we show here that it is not sufficient for WWP2 disinhibition. The three PY motifs of NDFIP2 are clustered within a region spanning amino acids 150 to 186. Mund and Pelham found that a 52 amino acid peptide of NDFIP2, including the three PY motifs, was able to disinhibit the related NEDD4 ligase Itch (11). All three PY elements were shown to contribute to activity, and it was concluded that higher avidity of multiple PY–WW interactions is important for this (11). Our results on WWP2 disinhibition with PY point mutants of NDFIP2 follow the same pattern, but while the single PY3 and double PY1/PY2 point mutants had reduced interaction with WWP2, the interaction of single PY1 and PY2 point mutants with WWP2 was similar to that of the wild-type protein. Furthermore, both single PY1 and PY2 point mutants were markedly reduced in their ability to disinhibit WWP2, suggesting that PY1 and PY2 enable catalytic disinhibition, in addition to their partially redundant roles in binding WWP2. Another intriguing result from experiments with NDFIP2 was that despite its three PY motifs, full-length NDFIP2 only bound to truncations of WWP2 containing the WW2 domain. Perhaps an initial strong interaction between WW2 and PY3 facilitates subsequent weaker (and undetectable by methods used here) interactions between PY1/PY2 and other WW domains. Structural and other biophysical/molecular dynamic studies are now required to demonstrate these WW-PY interactions. These may also help explain how TMEM127 and NDFIP2 interact with the same WW2 domain yet differ with respect to disinhibition of WWP2.

That TMEM127, SUSD6 and NDFIP2 are likely to interact with the WW2 domain of WWP2 suggests an important function for this domain. Structural work has shown that WW2 overlies a region called the hinge-loop region, which provides a point of flexibility between the N- and C-lobes of the HECT domain and is required for the ubiquitin transferase activity of the HECT domain (10, 28). Therefore, binding of an Ndfip to the WW2 domain might contribute to disinhibition by preventing interactions between WW2 and this region of the HECT domain.

Previous work has shown that WWP1, WWP2, and Itch are autoinhibited by interactions between the HECT domain and the WW domains or regions between the WW domains (10, 29). In contrast, other NEDD4-like E3 ubiquitin ligases, such as NEDD4 and Smurf, are held in an autoinhibitory state through C2-HECT domain interactions (12, 27). It has been shown recently that C2 domain-mediated autoinhibition of Nedd4L ligase can be relieved by membrane curvature induced by a Bin-Amphiphysin-Rsv (BAR) domain protein, FCHO2, through an interaction between the C2 domain and the membrane (14). In contrast to previous work assessing the contribution of the C2 domain to WWP2 autoinhibition *in vitro* (10, 27), our assay assessed WWP2 disinhibition in the presence of known and unknown endogenous Ndfips and other factors, such as membrane association, that are known to contribute to WWP2 disinhibition. These factors might account for the differences between our results and those of Chen *et al.* (2017) (10). Our work shows that the C2 domain is required for autoinhibition of WWP2 and suggests that through its interaction with SteD, the autoinhibitory effect of the C2 domain is lost. This strongly suggests that there is an interaction between membrane-bound SteD and the C2 domain, which is allosteric or initiates other downstream events, such as protein aggregation, resulting in the release of WWP2 ligase autoinhibition. Therefore, there appear to be at least two inhibitory interactions, involving both WW and C2 domains, that contribute to WWP2 autoinhibition.

Disinhibition of WWP2 by TMEM127 and SteD has similarities to disinhibition by the eukaryotic protein Dvl2. Dvl2 has a single PPxY motif that contributes to Dvl2-WWP2 binding, but Dvl2 also interacts with the C2 domain of WWP2 *via* its Dishevelled, Egl-10, and Pleckstrin (DEP) domain. Furthermore, the DEP domain also contributes to the disinhibition of WWP2 (21). Although there are no sequence similarities between the DEP domain of Dvl2 and SteD, it is striking that Dvl2 interacts with WWP2 through a PPxY motif together with a C2-interacting/activating domain. Therefore, SteD and TMEM127 might be functionally analogous (as a pair) to Dvl2.

NDFIP1 and NDFIP2 were the first adaptors shown to disinhibit WWP2 through an interaction *via* their three PY motifs (11). These could be considered canonical Ndfips. However, our findings, along with previous work (21) show that Ndfips are more diverse in the way in which they interact with and disinhibit WWP2. The non-canonical Ndfips include (a) the cytoplasmic Dvl2 with its single PPxY motif, requirement for polymerization, and interaction with the C2 domain (21); and those described here: (b) the SUSD6/TMEM127 endogenous co-activating pair, both containing PY motifs; and (c) the SteD/TMEM127 co-activating pair with a single PPxY motif of TMEM127 and C2-interacting properties of SteD (Fig. 6*C*). Further structural work is now required to provide deeper mechanistic insights into the binding and disinhibition of WWP2 by these proteins.

## Experimental procedures

**Table:** 

Reagents and tools table		
Reagent/resource	Reference or source	Identifier or catalog number
Experimental model		
Mel JuSo cells	Holden group (16)	RRID: CVCL_1403
Mel JuSo cells *TMEM127*^*−/−*^	(16)	
HEK293T	Holden Group (16)	RRID: CVCL_0063
HEK293T *TMEM127*^*−/−*^	This study	
*Salmonella enterica* serovar Typhimurium 12023	NCTC	NCTC 12023
*Salmonella enterica* serovar Typhimurium 12023 + pFCcGi	(16)	
Recombinant DNA		
M6P-FLAG-TMEM127	(16)	
M6P-FLAG-TMEM127 Y236A	(16)	
pHis_6_-Ubi	Gift from Pelham group	
M6P-FLAG-TMEM127 Y220A	This work	
M6P-FLAG-TMEM127 Y224A	This work	
M6P-FLAG-TMEM127 Y220A;Y224A	This work	
M6P-FLAG-TMEM127 Y220A;Y236A	This work	
M6P-FLAG-TMEM127 Y224A;Y236A	This work	
M6P-FLAG-TMEM127 Y220A;Y224A;Y236A	This work	
M6P-FLAG-TMEM127 Ala1	This work	
M6P-FLAG-TMEM127 Ala2	This work	
M6P-FLAG-TMEM127 Ala3	This work	
M6P-FLAG-TMEM127 Ala4	This work	
M6P-FLAG-TMEM127 Ala5	This work	
M6P-FLAG-TMEM127 Ala6	This work	
M6P-FLAG-TMEM127 Ala7	This work	
M6P-FLAG-TMEM127 Ala8	This work	
M6P-FLAG-TMEM127 Ala9	This work	
M6P-FLAG-TMEM127 Ala10	This work	
M6P-FLAG-TMEM127 Ala11	This work	
M6P-FLAG-TMEM127 Ala12	This work	
M6P-FLAG-TMEM127 Ala13	This work	
M6P-FLAG-TMEM127 Ala14	This work	
M6P-FLAG-TMEM127 Ala15	This work	
M6P-FLAG-TMEM127 Ala16	This work	
pTCMV-GFP-SteD	(26)	
pTCMV-GFP-SseG	(26)	
pTCMV-GFP-SteD 1-41	(26)	
pTCMV-GFP-SteD 37-111	(26)	
pTCMV-GFP-SteD 37-102	(26)	
pHA-WWP2 WT	(11)	
pHA-WWP2 C383A	(11)	
pHA-WWP2 1-171	This work	
pHA-WWP2 171-870	This work	
pHA-WWP2 362-870	This work	
pHA-WWP2 391-870	This work	
pHA-WWP2 485-870	This work	
pHA-WWP2 1-335	This work	
pHA-WWP2 1-363	This work	
pHA-WWP2 1-437	This work	
pHA-WWP2 1-477	This work	
pHA-WWP2 171-477	This work	
pUC-GW-Flag-NDFIP2	Vector backbone: pUC-GW-Amp Backbone manufacturer: GeneWiz Restriction sites: HindIII and NotI Purchased from Azenta	Vector backbone Map: https://cdn2.hubspot.net/hubfs/3478602/GENEWIZ_pUC-GW_Plasmid_Map.pdf
pTCMV-FLAG-NDFIP2	This work	
pTCMV-NDFIP2 Y151A	This work	
pTCMV-NDFIP2 Y177A	This work	
pTCMV-NDFIP2 Y186A	This work	
pTCMV-NDFIP2 Y151A;Y177A	This work	
pTCMV-NDFIP2 Y151;186AA	This work	
pTCMV-NDFIP2 Y177A;Y186A	This work	
pTCMV-FLAG-NDFIP2 Y151A;Y177A;Y186A	This work	
pUC-GW-SUSD6-FLAG	Vector backbone: pUC-GW-Amp Backbone manufacturer: GeneWiz Restriction sites: HindIII and NotI flanking SUSD6-FLAG Purchased from Azenta	Vector backbone Map: https://cdn2.hubspot.net/hubfs/3478602/GENEWIZ_pUC-GW_Plasmid_Map.pdf
pTCMV- SUSD6-FLAG	This work	
pTCMV-SUSD6-FLAG Y177A	This work	
pX330-U6-Chimeric_BB-CBh-hSpCas9	Gift from Teresa Thurston	(30)
Oligonucleotides and sequence-based reagents		
For mutant generation		
Primer sequence (5′-3′)	Primer use	
AACCGTCTCACATGGCCGATTACAAGGATGACGAC	Primer A for M6P-FLAG TMEM127 mutants	
AACCGTCTCAGGCCGCTTAGGGTGTGTAAGCAGGG	Primer A for M6P-FLAG TMEM127	
CCTCATATTCCGCCGGGGCGGG	Primer B M6P-FLAG-TMEM127 Y220A and M6P-FLAG-TMEM127 Y220A;Y236A	
CCCGCCCCGGCGGAATATGAGG	Primer C M6P-FLAG-TMEM127 Y220A and M6P-FLAG-TMEM127 Y220A;Y236A	
CCCTACCCGGCGGAAGCTGAGG	Primer B M6P-FLAG-TMEM127 Y224A and M6P-FLAG-TMEM127 Y224A;Y236A	
CCTCAGCTTCCGCCGGGTAGGG	Primer C M6P-FLAG-TMEM127 Y224A and M6P-FLAG-TMEM127 Y224A;Y236A	
CCCGCCCCGGCGGAAGCTGAGG	Primer B M6P-FLAG-TMEM127 Y220A;Y224A and M6P-FLAG-TMEM127 Y220A;Y224A;Y236A	
CCTCAGCTTCCGCCGGGGCGGG	Primer C M6P-FLAG-TMEM127 Y220A;Y224A and M6P-FLAG-TMEM127 Y220A;Y224A;Y236A	
AACCGTCTCAGGCCGCTTAGGGTGTGGCAGCAGGGGGTGGCTGGAA	Primer D M6P-FLAG-TMEM127 Y236A	
TTAAGCACGTCTCTGCAGCAGCAGCAGCAGCAGGGCTGCCCGGCGGG	Primer C M6P-FLAG-TMEM127 Ala1	
TTAAGCACGTCTCTCTGCCATTCCAGCCTTATCGTCG	Primer B M6P-FLAG-TMEM127 Ala1	
TTAAGCACGTCTCTCAGCAGCAGCACGCCGGCGGAGGAGCCCGGG	Primer C M6P-FLAG-TMEM127 Ala2	
TTAAGCACGTCTCTGCTGCTGCTGCGCCTCCGGGGGCGTACATTC	Primer B M6P-FLAG-TMEM127 Ala2	
TTAAGCACGTCTCTCAGCAGCAAGCCCGGGAGGCAGCGCTCTG	Primer C M6P-FLAG-TMEM127 Ala3	
TTAAGCACGTCTCTGCTGCTGCCCCGCCGGGCAGCCCTGCGC	Primer B M6P-FLAG-TMEM127 Ala3	
TTAAGCACGTCTCTCAGCAGCAGCACTGCCCAAGCAGCCGGAGCG	Primer C M6P-FLAG-TMEM127 Ala4	
TTAAGCACGTCTCTGCTGCTGCTGCCCTCCGCCGGCGCCCGCCGG	Primer B M6P-FLAG-TMEM127 Ala4	
TTAAGCACGTCTCTCGGCAGCACCGGAGCGTAGCCTGGCCTCG	Primer C M6P-FLAG-TMEM127 Ala5	
TTAAGCACGTCTCCGCCGCCGCAGCGCTGCCTCCCGGGCTCCTCC	Primer B M6P-FLAG-TMEM127 Ala5	
TTAAGCACGTCTCGCTGCAGCACTGGCCTCGGCCCTGCCTGG	Primer C M6P-FLAG-TMEM127 Ala6	
TCTCGCACGTCTCCGCAGCGGCCTGCTTGGGCAGAGCGCTGC	Primer B M6P-FLAG-TMEM127 Ala6	
TTAAGCACGTCTCTCAGCAGCAGCAAAGATCACTCGTCGCTATGC	Primer M6P-FLAG-TMEM127 Ala7	
TTAAGCACGTCTCCGCTGCAGCTGCCCCAAAGACATCCAGAAGG	Primer M6P-FLAG-TMEM127 Ala7	
TTAAGCACGTCTCTGCTGCAGCATATGCCTTCGCCCATATCCT	Primer C M6P-FLAG-TMEM127 Ala8	
TCTCGCACGTCTCGCAGCGGCAGCCAGAGCAGGATGCTTCGGCC	Primer B M6P-FLAG-TMEM127 Ala8	
TTAAGCACGTCTCGGCCGCAGCAGAGGAAGAGGAGCAGGCGCT	Primer C M6P-FLAG-TMEM127 Ala9	
TCTACTGCGTCTCGCGGCGGCTGCCAGGAGGTTGGCTGCCGTGG	Primer B M6P-FLAG-TMEM127 Ala9	
ATATCGTCGTCTCGCCGCAGCACAGGCGCTGGAGCTGCTCTC	Primer C M6P-FLAG-TMEM127 Ala10	
TTAAGCACGTCTCCGCGGCCGCTGTGGGGTAGTGGCGCAGGA	Primer B M6P-FLAG-TMEM127 Ala10	
TTAAGCACGTCTCTCAGCAGCAGCATCAGAGATGGAAGAGAACGA	Primer C M6P-FLAG-TMEM127 Ala11	
AATTGTCCGTCTCGGCTGCTGCTGCCTCCTCTTCCTCTGTGGGGT	Primer B M6P-FLAG-TMEM127 Ala11	
TTAAGCACGTCTCTGCCGCAGCAAACGAGCCCTACCCGGCGGA	Primer C M6P-FLAG-TMEM127 Ala12	
TTAAGCACGTCTCCCGGCGGCGGCGAGCAGCTCCAGCGCCTGCT	Primer B M6P-FLAG-TMEM127 Ala12	
TTAAGCACGTCTCGCCGCAGCACCGGCGGAATATGAGGTCAT	Primer C M6P-FLAG-TMEM127 Ala13	
TTAAGCACGTCTCCGCGGCCGCCTCTTCCATCTCTGAGAGCA	Primer B M6P-FLAG-TMEM127 Ala13	
TTAAGCACGTCTCTCAGCAGCAGCAATCAACCAGTTCCAGCCACC	Primer C M6P-FLAG-TMEM127 Ala14	
TTAAGCACGTCTCGGCTGCTGCTGCGTAGGGCTCGTTCTCTTCCA	Primer B M6P-FLAG-TMEM127 Ala14	
ATATCGTCGTCTCCGCCGCGGCCCCACCCCCTGCTTACACACC	Primer C M6P-FLAG-TMEM127 Ala15	
TTAAGCTCGTCTCTCCGCTGCTGCGACCTCATATTCCGCCGGGT	Primer B M6P-FLAG-TMEM127 Ala15	
GGGAGAGGGGCGGCCGCTTATGCTGCTGCTGCTGCTGCTGCCTGGAACTGGTTGATGACCT	R primer M6P-FLAG-TMEM127 Ala16	
GGAACATAAGTAGCTCCAGAGAACCGGCAC	F pHA-WWP2 1-171	
GAGCTACTTATGTTCCACTGGAGTCTCTCG	R pHA-WWP2 1-171	
GATCCTCTAGAATGACAGCAGTAGCTCCAGAGAA	F pHA-WWP2 171-870	
GATCCTCTAGAATGACCGCGGAGTACGTGCGCAA	F pHA-WWP2 362-870	
GATCCTCTAGAATGCTCTACCAGTCTTCGAGTGCTTCGA	F pHA-WWP2 391-870	
GATCCTCTAGAATGCAAGGTTCCCCTGGTGCTTATGACC	F pHA-WWP2 485-870	
GCCATTCTAGAGGATCCGGCGTAAT	R pHA-WWP2 truncations	
GAGCGGCCCCTTCCTCCATAATGGGAAAAACGCACAG	F pHA-WWP2 truncation 1-335	
CTGTGCGTTTTTCCCATTATGGAGGAAGGGGCCGCTC	R pHA-WWP2 truncation 1-335	
CCTGGCAGCGTCCGACCTAAGAGTACGTGCGCAAC	F pHA-WWP2 truncation 1-363	
GTTGCGCACGTACTCTTAGGTCGGACGCTGCCAGG	R pHA-WWP2 truncation 1-363	
CAGTGGGAGGATCCCCGGTAACAGGGGATGATCCAGG	F pHA-WWP2 truncation 1-437	
CCTGGATCATCCCCTGTTACCGGGGATCCTCCCACTG	R pHA-WWP2 truncation 1-437	
ACCTTTAAGGATCCTCGCTAGGGGTTTGAGTCGGGGA	F pHA-WWP2 truncation 1-477	
TCCCCGACTCAAACCCCTAGCGAGGATCCTTAAAGGT	R pHA-WWP2 truncation 1-477	
CAAATGGGCGGTAGGCGTG	Primer A pTCMV-FLAG-NDFIP2 mutants & pTCMV-FLAG-SUSD6	
GGTTCAGGGGGAGGTGTGGG	Primer D pTCMV-FLAG-NDFIP2 mutants & pTCMV-FLAG-SUSD6	
AACCGTCTCAGCTAGTAGTATTACTGTGGAAGTAC	Primer C pTCMV-FLAG-NDFIP2 Y151A	
AACCGTCTCATAGCTGGTGGAGGGGAAGAGTCAGTTT	Primer B pTCMV -FLAG-NDFIP2 Y151A	
AACCGTCTCAGCTAGCGTTGCTACCTCTCTTCCTACAGCCGATGAAGCTGA	Primer C pTCMV -FLAG-NDFIP2 Y177A;Y186A and Y151A;Y177A;Y186A	
AACCGTCTCATAGCGGGAGGTGGCACGGGATAAAACT	Primer B pTCMV -FLAG-NDFIP2 Y177A;Y186A Y151A;Y177A;Y186A	
ATCCCGTGCCACCTCCCGCTAGCGTTGCTACCTCTCT	Primer C pTCMV -FLAG-NDFIP2 Y177A and Y151A;Y177A	
AGAGAGGTAGCAACGCTAGCGGGAGGTGGCACGGGAT	Primer B pTCMV -FLAG-NDFIP2 Y177A and Y151A;Y177A	
CTACCTCTCTTCCTACAGCCGATGAAGCTGAGAAGGCTA	Primer C pTCMV -FLAG-NDFIP2 Y186A and Y151A;Y186A	
TAGCCTTCTCAGCTTCATCGGCTGTAGGAAGAGAGGTAG	Primer B pTCMV -FLAG-NDFIP2 Y186A and Y151A;Y186A	
GTTGCACTACCATCAGCCGAGGAGGCTGTATAT	Primer C pTCMV-FLAG-SUSD6	
TACAGCCTCCTCGGCTGATGGTAGTGCAACCTG	Primer B pTCMV-FLAG-SUSD6	
Sequencing		
Primer sequence (5′-3′)	Primer use	
CAGAAGTTTTGCATTGACAAAGTT	F Primer for sequence WWP2 C/A mutant after cloning	
GAGCCCACCGCATCCCCAGCATG	R primer for sequencing pHA-WWP2, anneals to BGH PA signal (on pHA-WWP2 plasmid)	
CTGGTGATATTGTTGAGTCA	F primer for sequencing M4P and M6P Plasmids	
TAGACGGCATCGCAGCTTGGA	Forward primer for sequencing M4P and M6P Plasmids	
CAAATGGGCGGTAGGCGTG	F primer for sequencing pTCMV	
GGTTCAGGGGGAGGTGTGGG	R primer for sequencing pTCMV	
GGGATCCTCCCACTGGGTCGTG	R primer for sequencing from mid WWP2	
GTCAAGAGAGCTCCTCCTTGCAGAG	F primer for sequencing TMEM127 from gDNA after CRISPR with sgRNA1 pX330	
CCAGCTCCTGCCACCAGGTAGAAGC	R primer for sequencing TMEM127 from gDNA after CRISPR with sgRNA1 pX330	
GAGCGTAGCCTGGCCTCGGCCCTGC	F primer for sequencing TMEM127 from gDNA after CRISPR with sgRNA2 pX330	
TGGGCATGAACACCAGGCAGTTATG	R primer for sequencing TMEM127 from gDNA after CRISPR with sgRNA2 pX330	
CRISPR knockout		
Primer sequence (5′-3′)	Primer use	Reference
CACCGCTGCAGTGTGCCACCGTCAT	TMEM127 F sgRNA1 used for HEK cell KO using PX330 plasmid	(16)
AAACATGACGGTGGCACACTGCAGC	TMEM127 R sgRNA1 used for HEK cell KO using PX330 plasmid	(16)
CACCGGTGCACATAGCCCAACACGT	TMEM127 F sgRNA2 used for HEK cell KO using PX330 plasmid	(16)
AAACACGTGTTGGGCTATGTGCACC	TMEM127 R sgRNA2 used for HEK cell KO using PX330 plasmid	(16)
Chemicals, enzymes, and other reagents		
DpnI	NEB	R0176L
BsmBI-V2	NEB	R0739S
BbsI	NEB	R0539S
HindIII-HF	NEB	R3104S
NotI- HF	NEB	R3189S
NcoI-HF	NEB	R3193S
Foetal calf serum	Gibco, Life Technologies	10082147
Dulbecco’s modified eagle medium	Sigma	D5796
Lipofectamine 2000	Invitrogen	11668-019
Opti-MEM reduced serum medium	Thermo Fischer	11058021
Polybrene	Sigma	TR-1003-G
Hygromycin	Sigma	H3274
Gentamicin	Sigma	G1914
Puromycin	Merck	P8833
Tris	Sigma	T1503
SDS pellets	Merck	75746
Glycerol	Sigma	49767
β-mercaptoethanol	Gibco	21985023
Polyvinylidene fluoride Immobilon-P	Millipore	IPVH00010
Tween®-20	Sigma	P2287
Enhanced Chemiluminescence solution	GE Healthcare	RPN2134
Complete mini protease cocktail	Roche	04693159001
Triton-X-100	Sigma	T9284
T4 polynucleotide kinase	NEB	M0201S
GFP beads	Chromotek	gta-20
Anti-FLAG M2 affinity gel	Sigma	A2220
Anti-HA agarose	Pierce	26181
Anti-His Dynabeads	Thermo Fischer	10103D
NP-40 TERGITOL™ solution	Sigma	NP40S
Iodoacetamide	Sigma	I1149
N-ethylmaleimide	Sigma	04259
Wizard Genomic DNA Purification Kit	Promega	A1120
Anti-TMEM127	Bethyl labs	A303-450A
Anti-HA.11	Biolegend	901502
Anti-Actin	Sigma	A2066
Anti-GFP	Chromotek	3H9
Anti-FLAG	Sigma	F7425
Anti-Tubulin	Proteintech	1G7
L243 antibody (mouse anti-HLA-DR)	Sigma	SAB4700731
Donkey anti-mouse IgG Alexa647	Invitrogen	A-31571
Goat anti-rabbit IgG conjugated HRP	Dako	P044801
Goat anti-mouse IgG conjugated HRP	Dako	P0447
Goat anti-rat IgG conjugated HRP	Cell signalling	7077S
Software		
Image Lab software	Bio-Rad	
Prism 9	GraphPad	
FlowJo	BD Biosciences	
Illustrator	Adobe	
Benchling	Benchling [Biology Software]. (2023). Retrieved from https://benchling.com.	
Equipment		
Trans-Blot Turbo Transfer system	Bio-Rad	
ChemidocTM Touch Imaging System	Bio-Rad	
Fortessa flow cytometer	BD Biosciences	

### Methods

#### Plasmid construction

Mutations were created using the overlap-PCR method (31) or by one-step site-directed mutagenesis (32). Following one-step site-directed mutagenesis, the PCR product was then restricted with DpnI. The ligated or DpnI-restricted products were transformed into *E. coli* DH5α. Colonies were selected for colony PCR. Appropriate primers (see sequencing primers above) were combined with plasmids and sent for sequencing with Azenta. Sequence files were uploaded to Benchling.com and aligned with the wild-type sequence to determine whether the mutation was successful.

#### Cell culture, transfection, virus production, and generation of stable cell lines

Human Embryonic Kidney 293T (HEK293T) cells and human Mel JuSo cells were maintained in Dulbecco’s modified Eagle medium (DMEM) supplemented with 10% heat-inactivated foetal calf serum (FCS). Cells were incubated at 37 °C in 5% CO2. When new cell lines were being created, 50 U ml^−1^ of penicillin and 50 μg ml^−1^ streptomycin were added to the DMEM.

For transfections, HEK293T or Mel JuSo cells were seeded. 16 h later, cells were transfected with a total of 0.25 to 1 μg DNA plus 3 to 5 μl of lipofectamine 2000 in Opti-MEM reduced serum medium per well. Cells were then collected 16 to 20 h later. For virus production, HEK293T cells were co-transfected with lentiviral expression vector and packing plasmids VSVG and GagPol. The lentiviral expression vectors used were M6P encoding FLAG-TMEM127 (PY mutants and alanine mutants). The culture media was replaced 24 h post-transfection, and supernatant containing viruses was collected at 48 h post-transfection and filtered.

To generate Mel JuSo cells stably expressing FLAG-TMEM127 for alanine scanning mutagenesis, lentiviruses were added to Mel JuSo cells with polybrene (8 μg ml-1). At 48 h post-transduction, cells were selected for with hygromycin (800 μg ml-1).

#### CRISPR knock out of *TMEM127* using the PX330 plasmid

The PX330 plasmid encodes both the small guide RNA (sgRNA) and the Cas9 nuclease. The sgRNAs targeting the *TMEM127* gene were the same sequence used in a previous publication (16). The PX330 construct was then transfected into HEK293T cells. The sgRNA oligos were annealed and phosphorylated in one reaction step. The annealed oligos and PX330 plasmid were then restricted using BbsI, and the resultant products were incubated with T4 ligase. The ligated product was transformed into *E. coli* DH5α as described above. HEK293T cells were transfected with the TMEM127 sgRNA PX330 plasmid and selected for with puromycin (0.8 μg ml^−1^). Single cells were placed into a 96-well plate using the dilution method and grown on to create clonal populations. An aliquot of the cells was lysed, and protein levels of TMEM127 were assessed by SDS-PAGE and western blotting. Suitable cell lines were then selected for genome sequencing. Genomic DNA was extracted using the Wizard Genomic DNA Purification Kit. Regions surrounding the gRNA position within the *tmem127* gene were ligated into a pTCMV plasmid to allow sequencing of both alleles. Plasmids were sequenced and analyzed as outlined above.

#### Bacterial strains and infection

*S. enterica* serovar Typhimurium 12023 was used for infections. *E*. *coli* strain DH5α was used for the transformation of plasmids to produce bacterial stocks. Bacteria were grown in Luria-Bertani (LB) medium supplemented with carbenicillin (50 μg ml^−1^), kanamycin (50 μg ml^−1^), or chloramphenicol (30 μg ml^−1^) as appropriate.

*S*. Typhimurium strains were incubated overnight at 37 °C in a shaking incubator with the appropriate antibiotics. The overnight culture was diluted 1:33 in LB and incubated in a shaking incubator at 37 °C for 3.5 h before adding to Mel JuSo cells at an MOI of 1:100 for 30 min. Cells were washed twice and incubated with fresh DMEM containing gentamicin (100 μg ml^−1^) for 1 h. After 1 h, the media was exchanged with fresh media containing gentamicin (20 μg ml^−1^). Cells were processed at 20 h post-invasion.

#### Western blotting

Cells were lysed in 0.1% Triton. Cell lysates in SDS buffer were loaded and run on 8 to 12% polyacrylamide gels. Protein was transferred onto polyvinylidene fluoride (PVDF) membranes by semi-dry transfer using the Trans-Blot Turbo Transfer system. Membranes were blocked with 5% milk in Tris-buffered saline with 0.01% Tween (TBS-T) for 1 h at room temperature. Membranes were incubated overnight with the primary antibody in 5% milk in TBS-T at 4 °C. The following morning, membranes were washed three times in TBS-T for a total of 30 min, incubated with secondary antibody in 5% milk in TBS-T for 90 min and washed three times in TBS-T for a total of 30 min. Enhanced Chemiluminescence solution was then applied, and the signal was detected using a ChemidocTM Touch Imaging System. Specificity of antibodies was determined through either transfected compared to non-transfected cells or wild-type compared to knock-out cell lines.

#### Green fluorescent protein (GFP), FLAG, and hemagglutinin (HA) co-immunoprecipitation (IP)

Cells were lysed (lysis solution - 0.5% Triton-X-100, 150 mM NaCl, 50 mM Tris-Cl pH 7.4, 5 mM EDTA, 5% glycerol, complete mini protease cocktail) and then centrifuged at 16,000*g* for 10 min at 4 °C. Post-nuclear supernatant (PNS; input) was mixed with washed GFP beads, anti-FLAG gel or anti-HA agarose for 2 h at 4 °C on a 360° roller. Beads/agarose were then washed 3 to 5 times in wash buffer (0.5% Triton-X-100, 50 mM NaCl, 50 mM Tris-Cl pH 7.4, 5 mM EDTA, 5% glycerol). For the GFP IP after the final wash, beads were resuspended in SDS loading buffer. For the HA and FLAG IP, the gel or agarose was resuspended in SDS loading buffer without the reducing agent. All samples (input and immunoprecipitate) were boiled at 95 °C for 5 min before analysis by SDS-PAGE and immunoblots were carried out as outlined above.

#### His pull-down of His-tagged ubiquitin

HEK293T cells were co-transfected with plasmids encoding proteins of interest and a plasmid encoding His_6_-tagged ubiquitin (11, 33). 16 h later, cells were lysed in a denaturing lysis/wash buffer (8 M urea, 50 mM Tris pH 8, 2 mM N-ethylmaleimide, 10 mM IAA, 0.2% SDS, 0.5% NP40). The lysate was sonicated. 20 μl of Dynabeads per sample were washed twice in the lysis/wash buffer. The lysate was incubated with Dynabeads on a rolling incubator for 2 h at room temperature. Dynabeads were then washed three times in the lysis/wash buffer and finally resuspended in SDS loading buffer. The input and beads were then boiled at 95 °C for 5 min before analysis by SDS page and Western blotting.

#### Flow cytometry

Surface levels of mMHCII on Mel JuSo cells were measured following infection with *S*. Typhimurium 12023 expressing GFP. Mel JuSo cells were infected as outlined above. 20 h post-invasion, cells were detached from cell culture plates using 2 mM EDTA in PBS. All antibodies were diluted in FACS buffer (5% FCS and 1 mM EDTA in PBS). Cells were incubated with monoclonal L243 for human mMHCII for 30 min on ice, washed in cold PBS and then incubated with Alex Fluor 647 donkey anti-mouse for 30 min on ice. Cells were then resuspended in FACS buffer and taken for immediate analysis by flow cytometry. Data were acquired using a Fortessa flow cytometer; the fluorescence intensity of each fluorophore for each cell was measured. Data were analyzed using FlowJo v10 software. The geometric mean fluorescence intensity (gMFI) was then calculated. Surface levels of mMHCII were calculated as a gMFI of infected (GFP-positive) over gMFI of non-infected cells (GFP-negative).

#### Data analysis: Graphs and statistics

The graph was created, and flow cytometry data were analyzed using GraphPad Prism 9. Multiple values were all compared against each other for analysis of flow cytometry data using an Ordinary one-way ANOVA followed by Tukey’s multiple comparisons test.

## Data availability

All data are presented within this manuscript.

## Supporting information

This article contains supporting information (15).

## Conflict of interest

The authors declare that they do not have any conflicts of interest with the content of this article.
