# Supplementary material for: Mammalian and bacterial adaptors function as co-disinhibitory pairs to activate the E3 ubiquitin ligase WWP2
Source: J Biol Chem. 2025 Oct 22;301(12):110847. doi: 10.1016/j.jbc.2025.110847 (PMC12720080; doi:10.1016/j.jbc.2025.110847)
Supplement: Supporting Figures [file mmc1.pdf]

**Wild type**	140	ALETDSSPPP YSSITVEVPT TSDTEVYGEF YPVPPPYSVA TSLPTYDEAE	190
**PY1(Y151A)**	140	ALETDSSPPP ASSITVEVPT TSDTEVYGEF YPVPPPYSVA TSLPTYDEAE	190
**PY2 (Y177A)**	140	ALETDSSPPP YSSITVEVPT TSDTEVYGEF YPVPPPASVA TSLPTYDEAE	190
**PY3 (Y186A)**	140	ALETDSSPPP YSSITVEVPT TSDTEVYGEF YPVPPPYSVA TSLPTADEAE	190
**PY12 (YY151,177AA)**	140	ALETDSSPPP ASSITVEVPT TSDTEVYGEF YPVPPPASVA TSLPTYDEAE	190
**PY13 (YY151,186AA)**	140	ALETDSSPPP ASSITVEVPT TSDTEVYGEF YPVPPPYSVA TSLPTADEAE	190
**PY23 (YY177,186AA)**	140	ALETDSSPPP YSSITVEVPT TSDTEVYGEF YPVPPPASVA TSLPTADEAE	190
**PY123(YYY151,177,186AAA)**	140	ALETDSSPPP ASSITVEVPT TSDTEVYGEF YPVPPPASVA TSLPTADEAE	190

S1 NDFIP2

Amino acid sequence of NDFIP2 showing PY mutants highlighted in red.

A

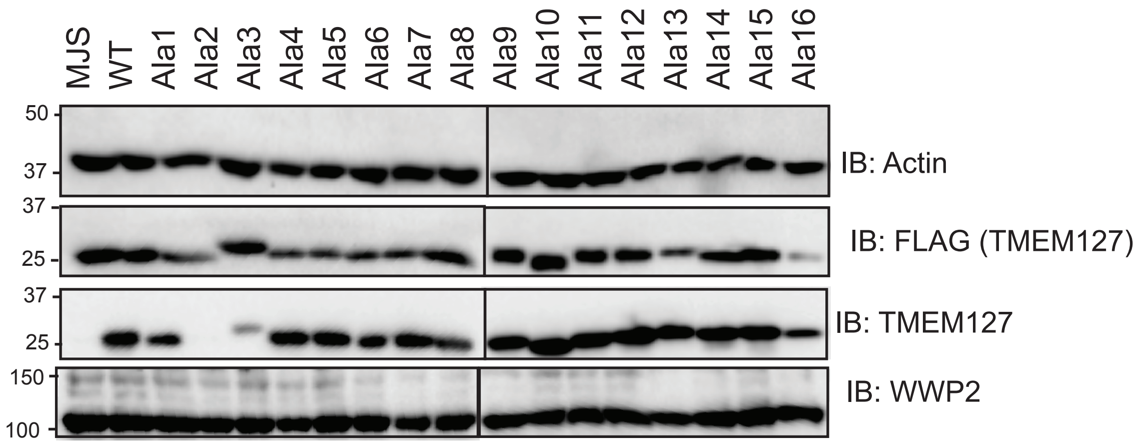

B

WT 210 LSEMEENEPY PAEYEVINQF QPPPAYTP 238
Y220A 210 LSEMEENEPA PAEYEVINQF QPPPAYTP 238
Y224A 210 LSEMEENEPY PAEAEVINQF QPPPAYTP 238
Y236A 210 LSEMEENEPY PAEYEVINQF QPPPAATP 238
Y220A;Y224A 210 LSEMEENEPA PAEAEVINQF QPPPAYTP 238
Y220A;Y236A 210 LSEMEENEPY PAEAEVINQF QPPPAATP 238
Y224A;Y236A 210 LSEMEENEPY PAEAEVINQF QPPPAATP 238
Y220A;Y224A;Y236A 210 LSEMEENEPA PAEAEVINQF QPPPAATP 238

C

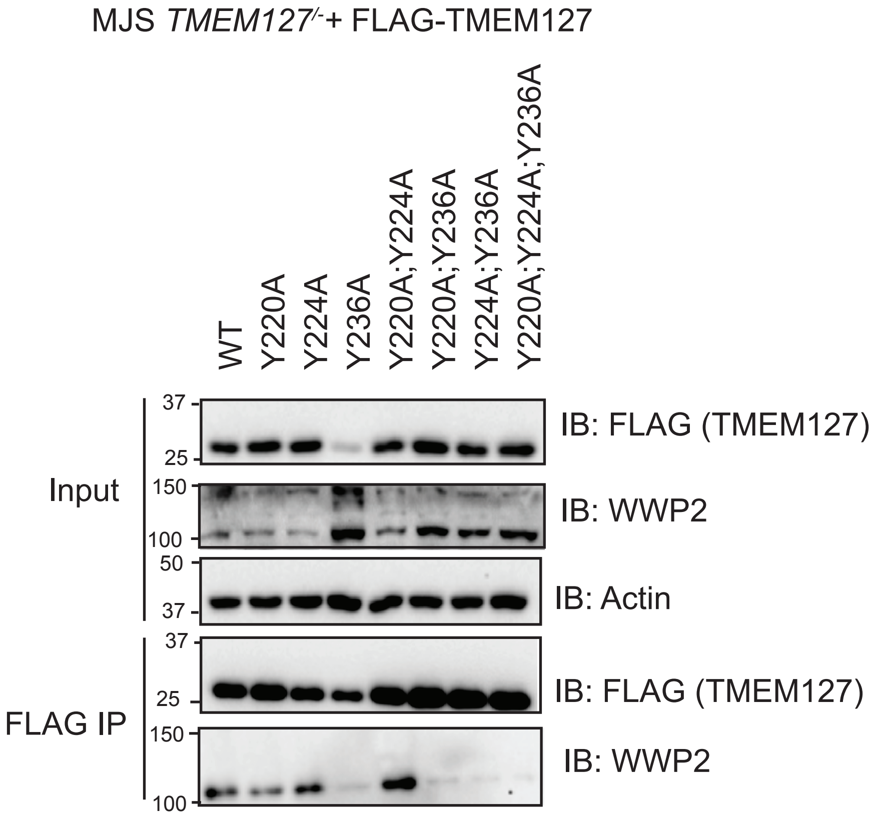

S2 TMEM127
A. Mel JuSo cells or mutant TMEM127 alanine mutant (as indicated) cell line levels of FLAG-TMEM127 as assessed by immunoblotting. B. Amino acid sequence of TMEM127 and PY mutants created from 210 to 238 with tyrosines within possible PY motifs indicated in red and mutations created indicated in orange. C. FLAG immunoprecipitation of TMEM127 PY mutant cell line (as indicated) and analysis of immunoprecipitate by immunoblotting. The blot shown is representative of the three independent experiments.

A

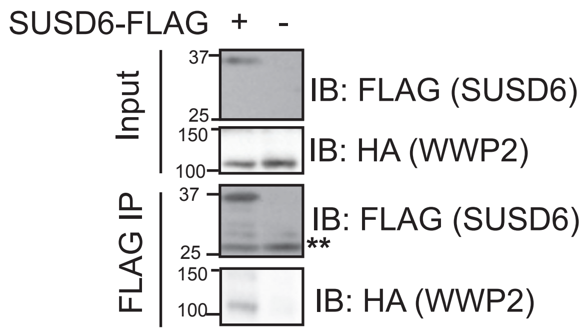

B

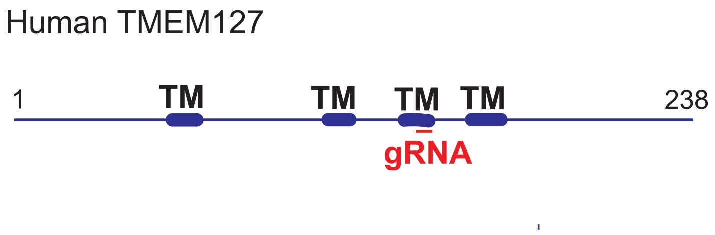

C

tmem127 allele 1
4bp deletion at 11895

TTCTGCAGTGTGCCA|~~CCGT~~|CATTGGCTTTTCTTATTGGGCTTCTGAACTCATCTTGGC
CCAGCAGCAGCAGCATAAGAAGTACCATGGATCCAGGTCTATGTCACCTTCGCCGT**TAG**

tmem127 allele 2
19bp deletion at 11889

GTTCTGCAGT|~~GTGCCACCGTCATTGGCTT~~|TTCTTATTGGGCTTCTGAACTCATCTTGGC
CCAGCAGCAGCAGCATAAGAAGTACCATGGATCCAGGTCTATGTCACCTTCGCCGT**TAG**

D

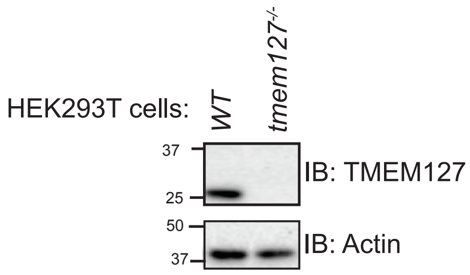

E

SteD truncation	Amino acids	Subcellular localisation
Full length	1-111	Normal (Golgi and endosomes)
N-terminal cytoplasmic region	1-41	Cytoplasmic
N-terminal truncation	37-111	Plasma membrane, Golgi and endosomes
C-terminal truncation	1-102	Normal (Golgi and endosomes)
N- & C-terminal truncation	37-102	Plasma membrane, Golgi and endosomes

F

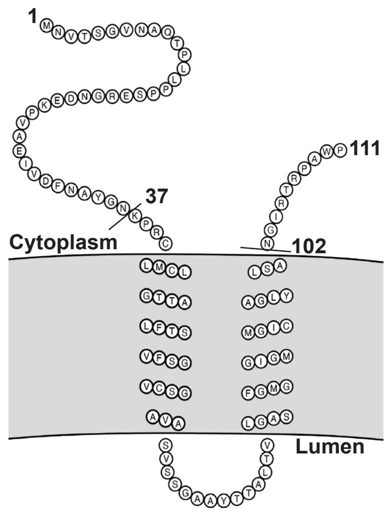

S3 SUSD6, generation of TMEM127$^{-/-}$ HEK293T cells and SteD

A. HEK293T cell transfection with/without SUSD6-FLAG and HA-WWP2 followed by FLAG-immunoprecipitation and immunoblot of immunoprecipitate (IP)**indicates light chain. **B**. Cartoon of TME127 with position of gRNA shown. **C.** HEK 293T pX330 *tmem127$^{-/-}$* sequencing results with deletions outline in blue and with strike through and premature stop codons in bold and red. **D.** Immunoblot of WT and TMEM127$^{-/-}$ cells. **E**. A summary of GFP-SteD truncations used in Figure 5, truncations previous published in (Bayer-Santos et al., 2016). **F**. Schematic of truncations of SteD in relation to SteD topology. IB, immunoblot.
